# Non-stem bladder cancer cell-derived extracellular vesicles promote cancer stem cell survival in response to chemotherapy

**DOI:** 10.1186/s13287-021-02600-6

**Published:** 2021-10-09

**Authors:** Wei-Min Chung, Ryan D. Molony, Yi-Fen Lee

**Affiliations:** 1 Department of Urology, University of Rochester Medical Center, 601 Elmwood Ave, Box 656, Rochester, NY 14642 USA; 2 Pathology and Laboratory Medicine, University of Rochester Medical Center, Rochester, NY USA; 3 Wilmot Cancer Institute, University of Rochester Medical Center, Rochester, NY USA

**Keywords:** Extracellular vesicles, Exosomes, Bladder cancer, Cancer stem cells, Non-stem cancer cells

## Abstract

**Background:**

Chemosenstive non-stem cancer cells (NSCCs) constitute the bulk of tumors and are considered as part of the cancer stem cell (CSC) niche in the tumor microenvironment (TME). Tumor-derived extracellular vesicles (EVs) mediate the communication between tumors and the TME. In this study, we sought to investigate the impacts of EVs released by NSCCs on the maintenance of CSC properties and chemoresistance.

**Methods:**

We employed murine MB49 bladder cancer (BC) sub-lines representing CSCs and NSCCs as a model system. Chemotherapy drugs were used to treat NSCCs in order to collect conditioned EVs. The impacts of NSCC-derived EVs on CSC progression were evaluated through sphere formation, cytotoxicity, migration, and invasion assays, and by analyzing surface marker expression on these BC cells. Differential proteomic analyses were conducted to identify cargo protein candidates involved in the EV-mediated communication between NSCCs and CSCs.

**Results:**

NSCC-derived EVs contained cargo proteins enriched in proteostasis-related functions, and significantly altered the development of CSCs such that they were more intrinsically chemoresistant, aggressive, and better able to undergo self-renewal.

**Conclusions:**

We thus identified a novel communication mechanism whereby NSCC-EVs can alter the relative fitness of CSCs to promote disease progression and the acquisition of chemoresistance.

**Supplementary Information:**

The online version contains supplementary material available at 10.1186/s13287-021-02600-6.

## Background

Bladder cancer (BC) is the fifth most commonly diagnosed malignancy in the United States, with approximately 73,510 diagnoses and 14,880 deaths in 2012 [1]. More than 70% of newly diagnosed BC cases are of the non-muscle invasive BC (NMIBC) subtype, and are confined to the epithelium and lamina propria [2, 3]. BC patients considered to be at a high risk of recurrence will be advised to undergo intravesical chemotherapy. However, two-thirds of patients will nonetheless experience tumor recurrence within five years following treatment, and this percentage rises to 88% after 15 years [4]. Approximately 25–30% of BC patients are diagnosed with muscle-invasive BC (MIBC), and initial treatment for these patients consists of potentially curative localized surgery and related therapies. Unfortunately, more than 50% of patients who undergo cystectomy for MIBC will relapse and succumb to metastatic disease. Chemotherapy has been considered to be effective as a means of suppressing BC progression and recurrence. However, BC is associated with high rates of chemoresistance and tumor relapse, and the underlying mechanisms of acquired chemoresistance remain largely unknown.

Cancer stem cells (CSCs) represent a small subpopulation of cells within tumors that are capable of undergoing self-renewal, tumorigenesis, and differentiation. These cells are often considered to be the driving force behind cancer heterogeneity, plasticity, and the acquisition of therapeutic resistance, thereby leading to disease recurrence and/or tumor metastasis. Analyses of molecular signatures associated with bladder CSCs have led to the identification of several makers and revealed their heterogeneity and intrinsic plasticity [5]. For instance, CSCs isolated from MIBCs exhibit higher expression levels of basal cell markers including CD44, P-cadherin, CK5, and CK14 [6–8]. In contrast, CSCs isolated from NMIBCs express higher levels of ALDH1A, CD133, Nestin, and CD90 [9, 10]. However, the lack of an in-depth understanding of the biology of bladder CSCs and their regulatory pathways has hindered their clinical application as prognostic markers and/or therapeutic targets. Therefore, the identification of the regulatory pathways that govern bladder CSC programs associated with the maintenance of stemness and differentiation during treatment and disease progression may be of significant clinical value.

The CSC niche refers to a spatial region within the tumor microenvironment that provides structural and functional interactions that contribute to the maintenance of CSC properties. CSCs and their niches constitute a dynamic ecosystem wherein CSC niche-derived signaling can influence CSC properties, and CSCs proactively remodel their niche to facilitate cancer progression. Extracellular vesicles (EVs) are a diverse group of membrane-enclosed particles secreted by cells into the extracellular space.There is increasing evidence that tumor-derived EVs play an integral role in cancer development and progression [11]. They transfer oncogenic cargos to recipient cells, consequently altering their behavior in ways that can support tumor growth and progression [12]. The molecular contents of EVs may reflect their cells of origin, making them useful diagnostic biomarkers. We have previously reported that several EV cargo proteins identified in the urine of BC patients can be analyzed to predict their clinical outcomes [13–15]. A number of studies have revealed that EVs play roles in communication between CSCs and their niche and that such bi-directional communication is critical for CSC self-renewal, differentiation, and survival, particularly in the context of chemotherapy [16, 17].

Non-stem cancer cells (NSCCs) form an integral part of the CSC niche, yet the functional roles of these NSCCs and EVs derived therefrom in supporting CSCs and facilitating the relative growth of cells with greater fitness under conditions of therapy-associated selective pressure are largely unknown. Here, we examined the roles of NSCC-derived EVs in the regulation of CSC properties and responses to chemotherapy using genetically matched murine MB49 BC cell sub-lines corresponding to these CSC and NSCC cell populations. Our results indicated that EVs released by NSCCs maintain CSC properties, enhance chemoresistance, and augment the aggressiveness of these cells.

## Methods

### Cell culture and reagents

The parental MB49 cells were a gift from Dr. Timothy Ratliff at Purdue University College of Veterinary Medicine and were cultured in RPMI 1640 (Thermo Fisher, USA) containing 10% fetal bovine serum (FBS) and 1% penicillin/streptomycin (Gibco, USA). The MB49 F1 and S2 sub-clones were established via the isolation of a single clone from MB49 primary cells as described previously [18]. Cisplatin (CDDP) was purchased from Sigma (USA). Gemcitabine was purchased from Millipore (USA).

### EV isolation and nanoparticle tracking analysis

Cells were grown at 50% confluence in RPMI containing 10% exosome-depleted FBS (prepared by overnight ultracentrifugation at 110,000 × g at 4ºC) for 48 h. To prepare EVs derived from CDDP- and gemcitabine-treated cells, cells were seeded, and after 16–18 h were treated for 48 h with sub-lethal doses of 5 µM CDDP or 5 nM gemcitabine. Cell culture supernatants were processed immediately after collection by serial centrifugation at 400 × g for 10 min and 15,500 × g for 30 min to remove cells and debris, and were then stored at − 80 °C. EVs were isolated from thawed samples by ultracentrifugation performed twice at 200,000 × g for 70 min at 4 °C, and the pellets were re-suspended in DPBS. Aggregates were removed from the samples by an additional 5-min centrifugation step at 15,500 × g. The final total protein concentration in each sample was measured with a Micro BCA assay (Thermo Fisher Scientific, #23,235), and EV samples were stored at -80 °C. Particle size distributions and concentrations in EV isolates were measured via a nanoparticle tracking analysis (NTA) performed using a NanoSight NS300 instrument (Malvern Instruments). Each sample was diluted 1:1000 in DPBS with negligible background signal, and five video files of 30 s each were recorded per sample. The concentrations of F1-EVs and S2-EVs were 3.67 ± 0.25 × 10^9^ particles/ml and 4.15 ± 0.27 × 10^9^ particles/ml, respectively.

### Cell viability assay

Cell viability was determined by methylthiazolyldiphenyl-tetrazolium bromide (MTT) assay. Briefly, 7 × 10^3^ MB49 cells (parental, F1, and S2 sub-clones) were seeded in 96-well plates in 100 µl volumes. Cells were treated with appropriate chemotherapy drugs for 48 h, after which an MTT assay was performed by adding MTT solution (1.25 mg/ml in RPMI) to these cells at a 1:1 ratio for 2 h at 37 °C. Absorbance associated with MTT-derived formazan was measured spectrophotometrically at 570 nm.

### Clonogenic assay

Appropriate cells were seeded at a density of 750 cells/well in 6-well plates in RPMI containing 10% FBS, and were allowed to grow into colonies. Cells were treated with the indicated concentrations of CDDP (DMSO, 0.625, 1.25, 2.5, 5, 10 µM) and gemcitabine (ddH_2_O, 0.25, 0.5, 1, 2, 4 nM for parental and F1 cells; ddH_2_O, 1.25, 2.5, 5, 10, 20 nM for S2 cells) for 8 days. Colonies were washed with PBS, fixed with 10% methanol/ 10% acetic acid for 10 min, and were stained with 0.4% crystal violet for 1 h. Colonies were then photographed and counted.

### Western blotting

Whole-cell lysates and EV samples were separated via 10% SDS-PAGE, and Western blotting was conducted as previously described [19]. The following antibodies were used: a mAb against TSG101 (1:1000, Santa Cruz Biotechnology, sc-794), a mAb against CD9 (1:1000 System Bioscience EXOAB-CD9A-1), a mAb against BiP (1:1000; catalog no. 3177, Cell Signaling); a mAb against PERK (1:1000; catalog no. 5683, Cell Signaling), and a mAb against GAPDH (1:10 K; Santa Cruz Biotechnology, sc-32233). Protein bands were detected with an ECL chemiluminescence kit (Millipore, USA), and were imaged with a ChemiDoc ™ XRS + instrument (BioRad, USA).

### Total RNA isolation and cDNA synthesis

RNA was extracted from MB49 cells as reported previously [18]. Briefly, cells that had reached 80–90% confluence in 100-mm dishes were lysed with 1 ml Trizol (Invitrogen, USA). Phenol/chloroform was then added, and RNA-rich layers were separated by centrifugation. Soluble RNA was precipitated using 2-propanol. RNA was then rinsed with 75% ethanol and dissolved in RNase-free water. For first-strand cDNA synthesis, 1 μg of total RNA was used for reverse transcription PCR conducted with the iScript™ RT Supermix kit (BioRad, USA) as per the manufacturer’s instructions.

### Quantitative real-time PCR

A real-time detection system (BioRad) and the iQ™ SYBR Green Supermix (BioRad) were used according to the manufacturer’s instructions. Relative gene expression was determined by normalizing the expression level of the target gene to the expression level of a housekeeping gene (GAPDH). Threshold value (Ct) dynamics were used (2^−ΔΔCt^) for the quantitation of gene expression. The qRT-PCR primer sequences were as follows: mGAPDH forward 5’- AGGTCGGTGTGAAC GGATTTG-3’, reverse 5’- TGTAGACCATGTAG TTGAGGTCA-3’; mCD133 forward 5'-CGGGATCCGAAAAACTGATCTGT-3', reverse 5'-CCGCTCGAGTTACCTAGTTACTCTCTCC-3'; mALDH1a1 forward 5'-CTCCTGGCGTGGTAAACATT-3', reverse 5'-CCATGGTGTGCAAACTCAAC-3'; mCD44 forward 5’- GAC CTC TGC AAG GCT TTC AA-3’, reverse 5’- TCC GAT GCT CAG AGC TTT CTC-3’; mNanog forward 5'-CAGCTGTGTGTACTCAATGATAGATTT-3', reverse 5'-ACACCATTGCTATTCTTCGGCCAGTTG-3'; mOCT-4 forward 5'-TCAGCCAAACGACCATCTGC-3', reverse 5'-TTCTCCAGGTTGCCTCTCAC-3'; mTWIST forward 5’-GGACAAGCTGAGCA AGATTCA -3’, reverse 5’-CGGAGAAGGCGTAG CTGAG -3’ [18].

### Sphere formation assay

Sphere formation assays were performed as reported previously [20]. MB49 cells were maintained in serum-free CSC culture medium: RPMI supplemented with 20 ng/mL mouse recombinant epidermal growth factor (mEGF) (PeproTech, USA), 20 ng/mL mouse recombinant basic fibroblast growth factor (mbFGF) (PeproTech, USA), 20 ng/mL mouse leukemia inhibitory factor (mLIF) (R&D, MN, USA), B-27 (Gibco, USA), and 0.4% BSA (Sigma, USA). An S2 single-cell suspension maintained in CSC medium was subsequently cultured in ultra-low attachment 6-well plates (Thermo Fisher, USA) at a density of < 5,000 cells/well for 10 days. Spheres that had formed during this period were recorded and colonies with a diameter greater than 50 μm were counted and photographed with a phase-contrast fluorescence microscope (Nikon, ECLIPSE 80i).

### Transwell migration/invasion assay

Transwell polycarbonate membrane inserts with an 8 μm pore size (Corning, #3422) were used for all migration and invasion experiments. For migration assays, 2.5 × 10^5^ cells were re-suspended in 200 µl of serum-free RPMI medium in the upper chamber of these Transwell inserts, while the lower chamber was filled with 650 µl of RPMI containing 10% FBS. Cells were allowed to migrate for 48 h. For invasion assays, these Transwell chambers were first coated with BD Matrigel™ Basement Membrane Matrix (BD Biosciences), after which 2.5 × 10^5^ cells were added to the upper chamber as above and incubated for 72 h. After appropriate incubation periods, inserts were collected and migratory/invasive cells were fixed with 4% paraformaldehyde, stained with 0.05% crystal violet, photographed, and counted.

Proteomic analyses EVs isolated from MB49 F1 and S2 cells were subjected to a series of sample preparation steps as previously described [21]. Digested peptide samples were frozen, dried, and re-suspended in 0.1% trifluoroacetic acid prior to analysis. Next, 1 µg of peptides were injected onto a C18 column with an Easy nLC-1200 HPLC, connected to a Fusion Lumos Tribrid mass spectrometer. Raw data was searched using the SEQUEST search engine within the Proteome Discoverer software platform (v2.4) using both the entire SwissProt database, as well as searches restricted to *mus musculus*. Trypsin was selected as the enzyme, allowing for up to 2 missed cleavages, with an MS1 mass tolerance of 10 ppm, and an MS2 mass tolerance of 0.6 Da. Carbamidomethyl was set as a fixed modification, while oxidation of methionine was set as a variable modification. The Minora node was used to determine relative protein abundance between samples using the Summed Abundance default settings. Percolator was used as the FDR calculator, filtering out peptides that had a q-value greater than 0.01.

### Statistical analysis

Data were compared between groups using the Student’s t-tests or one-way analyses of variance (ANOVAs) with Tukey’s multiple comparisons test as appropriate. All experiments were repeated at least three times, and a P-value < 0.05 was considered to indicate statistical significance. Statistical outcomes were labled as follows (ns: not significant; *P < 0.05; **P < 0.01; ***P < 0.001; ****P < 0.0001).

## Results

### MB49 sub-clones possess the properties of cancer stem cells and non-stem cancer cells

**Fig. 1:**
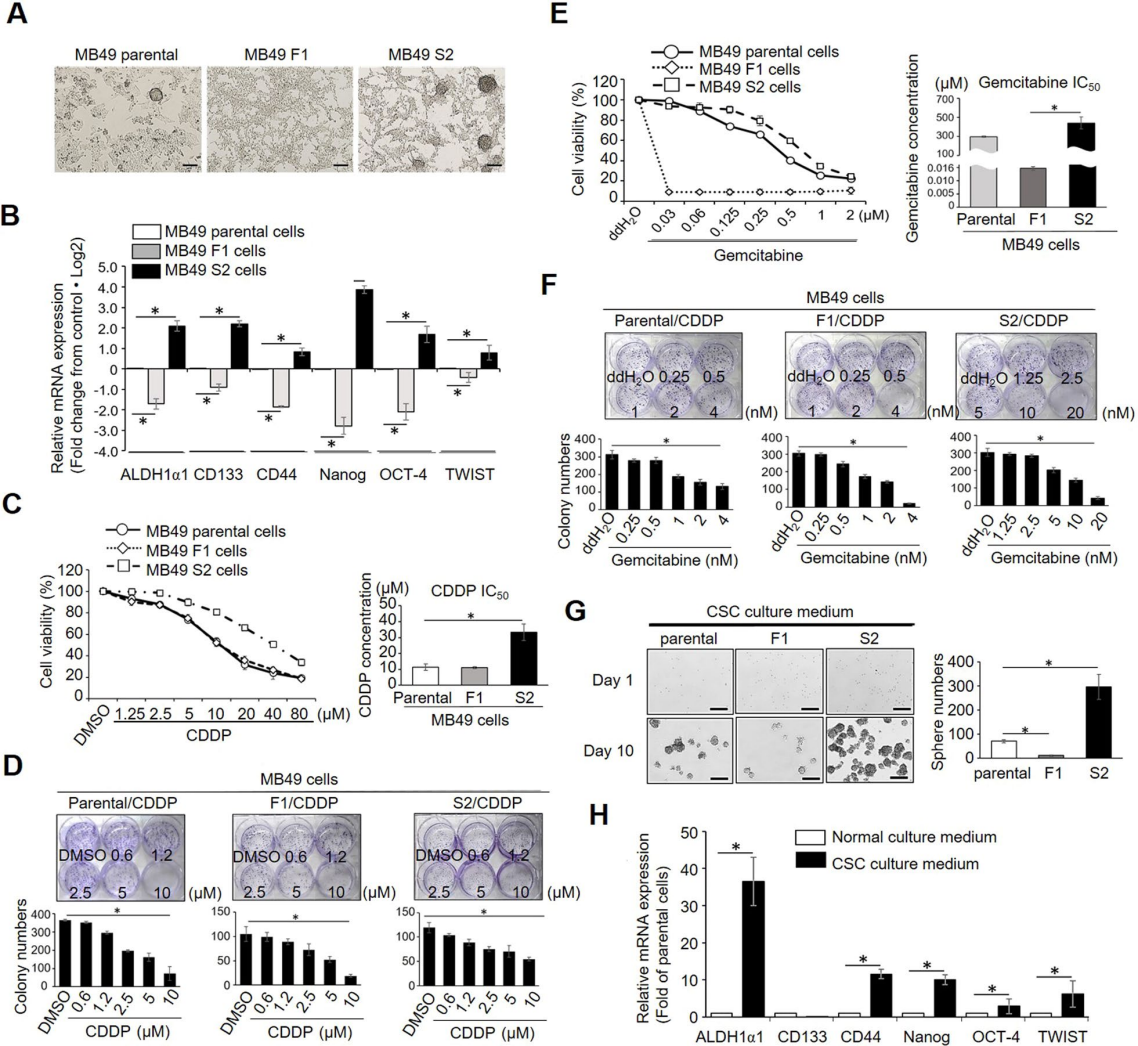
Characterization of the stemness properties of MB49 parental cells and MB49 F1 and S2 sub-clones. **a** Images showing differences in the morphology of MB49 parental, F1, and S2 cells. **b** CSC marker expression levels were compared among MB49 parental, F1, and S2 cells, with relative expression being normalized to MB49 parental cells. **c** MB49 cell sensitivity to CDDP and **e** gemcitabine was measured via MTT assay following treatment for 48 h with the indicated doses of these drugs (0, 0.03, 0.06, 0.125, 0.25, 0.5, 1, 2 µM). The absolute light absorbance at 540 nm was measured and normalized to vehicle control. **d** Determination of cell sensitivity to CDDP or **f** gemcitabine via clonogenic assay. Colonies were photographed and counted. **g** MB49 cell sphere formation assay. MB49 parental, F1 and S2 cells were cultured in serum-free CSC culture medium for 10 days and sphere images were taken on days 1 and 10 by phase-contrast microscope (50X). **h** Enhancement of CSC markers expression, as measured by qPCR, was observed when MB49 S2 were cultured in CSC medium. Relative expression levels were normalized to MB49 S2 cells in normal culture. Experiments were repeated independently at least three times. Data were compared via Student’s t-tests or one-way ANOVAs with Tukey’s multiple comparisons test, as appropriate. *P < 0.05; **P < 0.01; ***P < 0.001

To study the functional roles of NSCCs as a part of the CSC niche, we used two sub-clones derived from the murine MB49 bladder cancer cell line established in our laboratory [18]. They are MB49 S2 cells with stem-like properties and MB49 F1 that exhibit properties similar to those of NSCCs. The parental MB49 cells are a heterogeneous population within which a small portion of cells spontaneously form spheroids. The MB49 F1 cells grow as a monolayer, in contrast to MB49 S2 cells that form spheroids spontaneously under normal cell culture conditions (Fig. 1a). Molecular characterization of CSC markers revealed that MB49 S2 cells expressed higher levels of CSC markers than MB49 parental cells, whereas MB49 F1 cells displayed significantly reduced CSC marker expression (Fig. 1b). Chemoresistance is a hallmark of CSCs, and we thus compared the relative chemosensitivity of these cells through MTT and clonogenic assays. Cells were treated with CDDP and gemcitabine, two of the most commonly used chemotherapeutic drugs in BC, revealing that MB49 S2 cells were more resistant to CDDP (IC_50_: 33.2 ± 5.17 µM) relative to MB49 parental and MB49 F1 cells which exhibited an IC_50_ of approximately 11.03 µM (Fig. 1c) in an MTT assay. Similarly, These S2 cells were able to form more colonies under CDDP treatment conditions in a clonogenic assay relative to parental and F1 MB49 cells (Fig. 1d). Similar results were obtained following gemcitabine treatment, with MB49 S2 cells exhibiting increased gemcitabine resistance in both MTT and clonogenic assays. Intriguingly, MB49 F1 cells displayed extremely acute sensitivity to gemcitabine with an IC_50_ that was 18,000—30,000-fold lower than that of parental and MB49 S2 cells (Fig. 1e and f). Self-renewal ability, which is the essential defining characteristic of CSCs, was also compared between MB49 S2 and MB49 F1 cells. To test this, we cultured those cells for 10 days in low-attachment culture dishes with chemically defined CSC-culture medium proven to effectively maintain CSC properties under serum-free conditions during CSC expansion, and spheroid numbers were recorded. As expected, MB49 S2 cells formed more and larger spheroids than did MB49 parental cells, whereas no sizable spheroids were formed by MB49 F1 cells (Fig. 1g). The spheroids derived from MB49 S2 cells also exhibited enriched CSC marker expression (Fig. 1h), suggesting that this self-renewal process is enriched for MB49 S2 CSC populations. Taken together, these data offer multiple lines of evidence that MB49 S2 cells possess CSC-like properties including elevated CSC marker expression, chemoresistance, and self-renewal. In contrast, MB49 F1 cells more closely represent NSCCs as they exhibit low CSC markers level expression, are unable to form spheroids, and are highly sensitive to CDDP and gemcitabine. These genetically matched MB49 F1 and MB49 S2 cells thus provide a unique opportunity to study the interactions between NSCCs and CSCs via their EVs in order to understand how they influence CSC properties and cancer progression.

### NSCC-derived EVs regulate CSC function

**Fig. 2:**
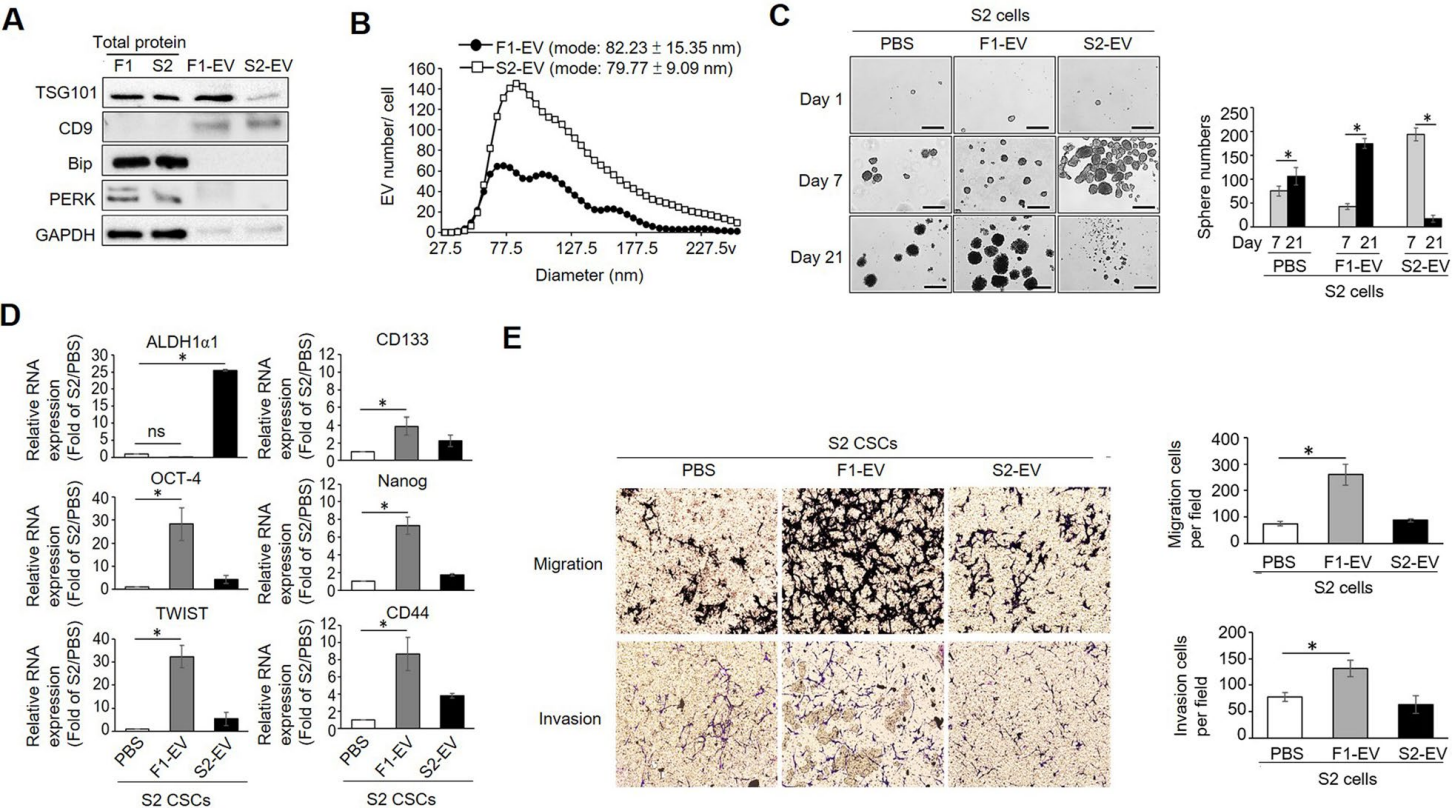
NSCC-derived EVs affect CSC properties. **a** EVs were purified from MB49 F1 and S2 cells, and their purity *(10 µg of total EVs proteins)* was confirmed by Western blotting for exosome markers (TSG101 and CD9) and ER-resident proteins (Bip and PERK) to exclude potential cytoplasmic contamination. **b** Determination of EV secretion rate in MB49 F1 and S2 cells by NTA. **c** The effect of EVs on MB49 S2 cell sphere formation was measured by seeding MB49 S2 cells (1000 cells/mL) in CSC culture medium and 10 µg/ml EVs were used to treat S2 CSCs for 21 days. Images of spheroid formation on days 1, 7, and 21 are shown (50x). Spheroid numbers were counted and the resultant quantitative data were plotted. **d** The expression of CSC markers in the S2 cells shown in C. PBS treatment was used as a normalization control, and relative expression was calculated via the 2^−ΔΔCt^ method. **e** F1-EVs promote MB49 S2 cell migration and invasion. S2 cells maintained in CSC medium were treated with F1-EVs, S2-EVs, or PBS for 10 days, cells were subjected to migration and invasion assays using 10% FBS as a chemoattractant. For migration assay, cells were incubated for 48–72 h. Experiments were repeated independently at least three times. Data were compared via Student’s t-tests or one-way ANOVAs with Tukey’s multiple comparisons test, as appropriate. *P < 0.05; **P < 0.01; ***P < 0.001

To study how NSCCs may influence the function of CSCs through the secretion of EVs, we purified EVs from MB49 F1 and MB49 S2 cells, and their quality and quantity were confirmed. As shown in Fig. 2a, EVs derived from MB49 F1 and S2 both expressed the exosome markers TSG101 and CD9 to differing degrees. In addition, we were able to detect the expression of two ER resident proteins, Bip and PERK, only in the cell lysates but not in EV samples, suggesting that there was no cytosolic protein contamination in EV samples. Moreover, nanoparticle tracking analysis (NTA) indicated that MB49 S2 cells release significantly more EVs than do MB49 F1 cells, but that there was no difference in EV size distributions between these two EV populations (F1-EV size: ~ 82 ± 15 nm; S2-EV size: ~ 79 ± 9 nm) (Fig. 2b). Then, we evaluated the impacts of EVs on the self-renewal abilities of MB49 S2 cells. We found that treating MB49 S2 with their own EVs accelerated their self-renewal, whereas no significant change in spheroid numbers was observed when MB49 S2 cells were treated with MB49 F1-EVs for 7 days. Unexpectedly, continued exposure of MB49 S2 cells to their own EVs for 21 days led to their premature death (Fig. 2c, left panel), whereas MB49 F1-EV treatment significantly increased spheroid numbers and size at day 21 (Fig. 2c, middle panel). These unexpected results might be caused by a higher proliferation rate in the S2EV-treated spheres that could result in high density when the culture period was extended to 21 days, which eventually leads to faster growth decline, consistent with the occurrence of apoptosis. To assess the molecular alterations induced by these F1-EVs, we measured CSC marker expression on S2-EV-treated spheroids, revealing that F1-EVs and S2-EVs induced distinct patterns of marker gene expression. S2-EVs promoted the expression of ALDH1α1, OCT-4, TWIST, and CD44, whereas F1-EVs induced CD44, CD133, OCT-4, Nanog, and TWIST upregulation (Fig. 2d). These findings suggest that EVs derived from MB49 F1 and MB49 S2 cells encapsulate specific cargo molecules that engage distinct signaling pathways in recipient CSCs.

Higher levels of CD44, CD133, and ALDH1α1 in bladder CSCs are known to be associated with BC progression and aggressiveness. To test whether F1-EV-treated (CD44 ^high^/CD133^high^) and S2-EV-treated (ALDH1α1^high^) MB49 S2 sphere cells exhibited more aggressive behaviors, we conducted migration and invasion assays. As shown in Fig. 2e, f1-EV-treated, but not S2-EV-treated MB49 S2 cells exhibited increased migration and invasion capacity as compared to PBS-treated MB49 S2 cells (Fig. 2e). Taken together, these data imply that EVs released by NSCCs can sustain CSC stemness and promote aggressive behaviors. To our knowledge, this is the first report to have illustrated a functional role for NSCCs in supporting CSC properties via the production of EVs.

### NSCC-derived EVs promote CSC survival under chemotherapy

**Fig. 3:**
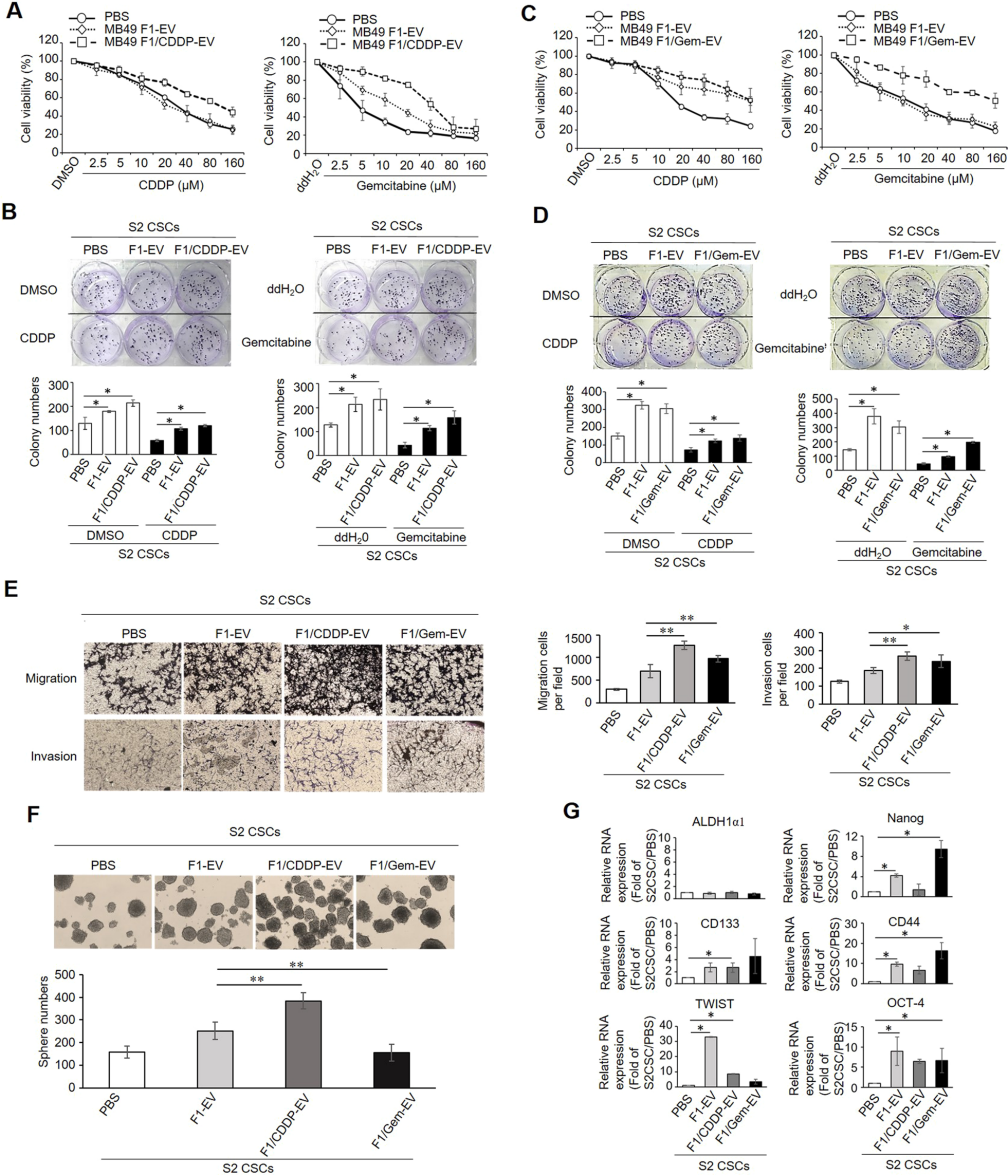
EVs derived from chemotherapy-treated NSCCs enhance CSC chemoresistance and progression. **a–d** Chemotherapy-induced F1-EVs promote the acquisition of chemoresistance in CSCs. S2 cells were pre-conditioned with naïve F1-EVs, F1/CDDP-EVs, or F1/Gem-EV for 10 days. Cells were subjected to chemotoxicity tests using CDDP (DMSO, 2.5, 5, 10, 20, 40, 80, 160 µM), and gemcitabine (ddH_2_O, 2.5, 5, 10, 20, 40, 80, 160 µM) for 48 h, after which MTT assays (**a** and **c**) and clonogenic assays (**b** and **d**) were performed. **e** Chemotherapy-induced F1-EVs enhance CSC invasion and mobility. S2 cells maintained in CSC medium were treated with F1/CDDP-EV, F1/Gem-EV, or PBS for 10 days. Cells were then collected and subjected to transwell migration and invasion assays. Representative images of cells in the lower chambers are shown, and cell numbers were quantified and plotted. **f** Chemotherapy-induced F1-EVs promote the self-renewal of CSCs. S2 cells maintained in CSC medium were treated with F1/CDDP-EV, F1/Gem-EV, or PBS control for 10 days. Single suspended cells (1000 cells/ml) were seeded in CSC culture medium without any additional treatment for 10 days. Spheroid forming ability was determined by counting the number of spheres. Representative images on day 10 were taken, and sphere numbers were counted and plotted. **g** Chemotherapy-induced F1-EVs alter CSC marker expression. CSC markers in the spheroids described in (**f**) were measured by quantitative real-time PCR. Data were normalized to PBS controls, and the 2^−ΔΔCt^ method was used to calculate relative gene expression. Experiments were repeated independently at least three times. Data were compared via Student’s t-tests or one-way ANOVAs with Tukey’s multiple comparisons test, as appropriate. *P < 0.05; **P < 0.01; ***P < 0.001

CSCs are known to exhibit higher rates of endogenous chemoresistance relative to NSCCs such that chemotherapy treatment eliminates the bulk of NSCC populations while leaving CSC populations relatively intact. As we found that EVs released by NSCCs can sustain CSC stemness and promote aggressive growth, we were interested in additionally assessing whether EVs released by MB49 F1 cells under chemotherapy conditions would have any impact on the survival of MB49 S2 cells. For these experiments, MB49 F1 cells were treated with a sub-lethal dose of CDDP (5 µM) or gemcitabine (5 nM), and EVs (naïve F1-EVs, F1/CDDP-EVs, and F1/Gem-EVs) were collected to test their impact on CSC function under chemotherapy treatment conditions. MB49 S2 cells were pre-conditioned with chemotherapy-treated F1-EVs or naïve EVs for 10 days in CSC culture medium, after which the cells were characterized for their chemo-sensitivity, migration, invasiveness, and self-renewal properties. We found that naïve F1-EVs, as well as F1/CDDP-EVs, were able to enhance the chemoresistance of MB49 S2 cells not only to CDDP but also to gemcitabine in both MTT (Fig. 3a) and clonogenic assays (Fig. 3b). We also found that both naïve F1-EVs and F1/CDDP-EVs were able to promote MB49 S2 colony formation, suggesting that F1-EVs contain cargo molecules that are involved in facilitating CSC division/self-renewal. Similar results were also seen in the gemcitabine group, where we found F1/Gem-EV treatment led MB49 S2 cells to acquire resistance to gemcitabine as well as to CDDP (Fig. 3c and d). Next, we tested whether these F1-EVs derived from chemotherapy-treated NSCCs were able to promote CSC aggressiveness. As expected, treating MB49 S2 cells with F1/CDDP-EVs and F1/Gem-EVs enhanced their migration and invasion capacity as compared to PBS control (Fig. 3e). Our data suggest an interesting scenario where, under chemotherapy treatment conditions, the majority of chemo-sensitive NSCCs will release their EVs and thereby promote the survival and aggressive growth of proximal CSCs, thus driving tumor progression. Next, we tested the impact of chemotherapy-induced F1-EVs on MB49 S2 cell self-renewal ability and found that F1/CDDP-EVs enhanced MB49 S2 spheroid numbers and size relative to PBS control treatment. Interestingly, F1/Gem-EV treatment enhanced the size of these spheres on average but not sphere numbers (Fig. 3f). Then, CSC markers expression on the F1/CDDP-EV- and F1/Gem-EV-treated MB49 S2 spheroids was analyzed. Consistent with the observed cellular phenotypes, we found that almost all CSC markers other than ALDH1α1 were elevated more substantially in F1-EV-treated S2 spheroids relative to PBS control-treated groups (Fig. 3g).

### MB49 FI-EVs are enriched for cargo proteins that regulate proteostasis

**Fig. 4:**
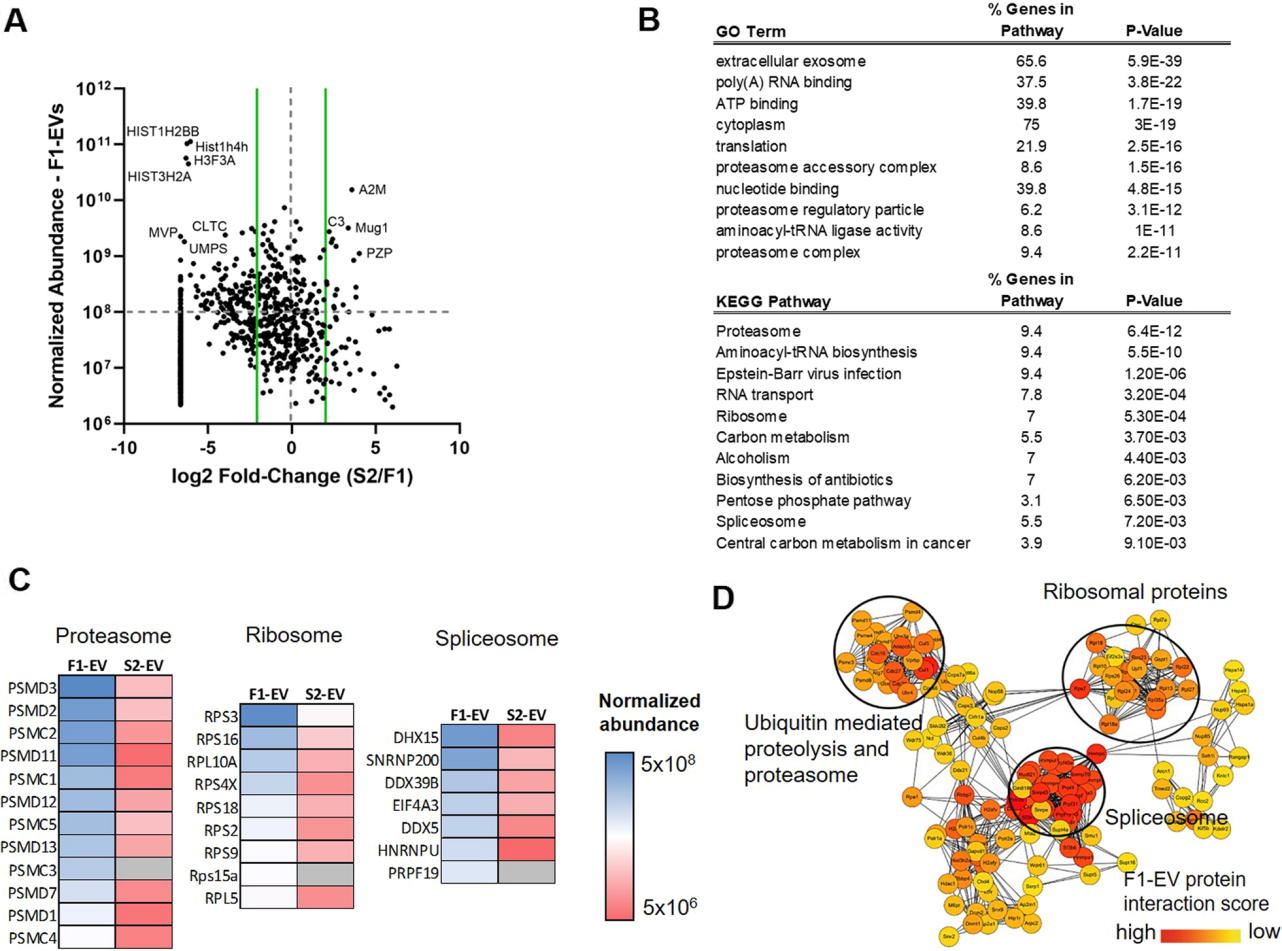
Proteomic analyses highlight potential functional roles for F1-EV cargo proteins. **a** Normalized abundance values for F1-EV and S2-EV cargo proteins were calculated following LC–MS/MS, and log2 fold-change (FC) values were calculated by comparing the relative abundance of a given protein in F1-EV and S2-EV samples. Green lines correspond to a |log2(FC)|≥ 2.0, and the horizontal gray line corresponds to the arbitrary threshold for highly abundant proteins used in GO/KEGG pathway analyses. **b** Top GO terms and KEGG pathways corresponding to highly abundant proteins that were preferentially enriched in F1-EVs (|log2(FC)|≥ 2.0; relative abundance ≥ 10^8^; n = 128), as calculated using the DAVID bioinformatics database **c** Heatmaps demonstrating the relative abundance of proteins in the Proteasome, Ribosome, and Spliceosome KEGG pathways in F1-EV and S2-EV samples. **d** Cytoscape functional analyses identifying three major functional protein clusters enriched in F1-EV cargo proteins: the ubiquitin-mediated proteolysis and the proteasome, spliceosome, and ribosomal proteins clusters. Colors correspond to relative protein expression levels, and proteins are clustered according to predicted functions. Darker colors indicate a higher level of expression/interaction relative to lighter colors

To gain insight into the potential functional mechanisms whereby F1-EVs support the maintenance and chemoresistance of CSC populations, we analyzed F1-EV and S2-EV cargo proteins via quantitative LC–MS/MS. This analysis revealed many proteins that were differentially abundant between these two EV types (Fig. 4a), including multiple histones and a range of cargos that were not detected in S2-EVs. GO and KEGG analyses of those proteins that were highly abundant and highly enriched in F1-EVs (normalized abundance > 10^8^, |log2(FC)|> 2) highlighted the close association between these cargo proteins and key proteostasis-related pathways (Fig. 4b). Notably, enriched terms were associated with ribosome function, translational activity, and proteasome-mediated protein degradation, suggesting that these F1-EVs may deliver these proteins to CSCs wherein they have the potential to buffer cellular responses to chemotherapy and other stressful stimuli by modulating intracellular protein dynamics. Protein functional clustering analyses further confirmed that these F1-EV-enriched cargo proteins could be grouped into three major interacting protein groups: proteins associated with ubiquitin-mediated proteolysis and the proteasome, spliceosome-related proteins, and ribosomal proteins (Fig. 4d). CSCs are known to exhibit relatively limited translational and proteolytic activity, yet must be able to respond to dynamic changes in the tumor environment by rapidly controlling the biogenesis and degradation of proteins. The horizontal transfer of F1-EV cargos that contain the protein synthesis/degradation machinery may thus be critical in shaping CSC responses. Indeed, F1-EVs contained high levels of a range of different 26S proteasome subunits, ribosomal proteins, and spliceosome-related factors that were largely absent or completely undetectable in S2-EVs (Fig. 4c), consistent with the limited translational throughput of and proteolytic potential of these CSCs. Interestingly, these CSC-derived EVs were instead primarily enriched for cargo proteins associated with the complement and coagulation cascades that were present at only low levels F1-EVs (Additional file 1: Fig. 1), although the functional implications of this finding are unclear and warrant further research. While functional studies will be necessary to confirm the mechanistic roles of these identified NSCC-derived EV cargo proteins in the maintenance of CSC cell populations, our data nonetheless align well with what is known regarding the regulation of proteostasis in these two cell populations and provide a valuable resource that will guide future research efforts to better understand this novel mode of cell–cell communication that is critical for the survival, maintenance, and differentiation of CSCs.

## Discussion

**Fig. 5:**
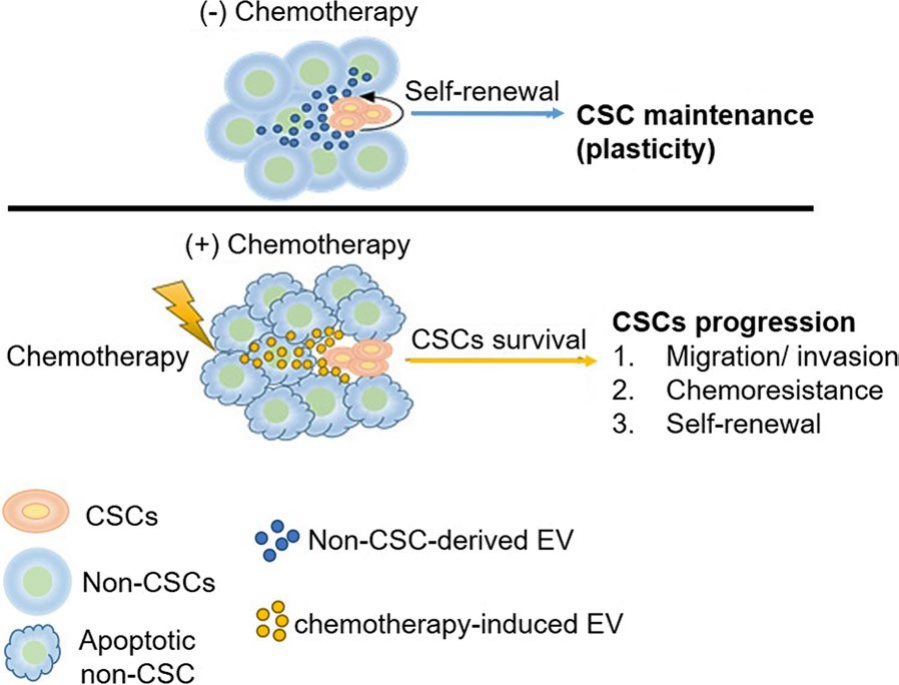
A schematic overview of the mechanisms whereby non-stem bladder cancer cell-derived extracellular vesicles promote cancer stem cell survival in response to chemotherapy

The eradication of CSCs remains a major challenge to cancer treatment owing to the specific characteristics of these cells that contribute to chemoresistance, relapse, and metastasis [22–24]. While growing numbers of studies have focused on identifying intrinsic factors that determine CSC properties, relatively little is understood regarding the complex influence of extrinsic factors released from the CSC niche on CSC properties, hindering efforts to effectively target CSCs [25–28]. As summarized in Fig. 5, in this study we found that EVs derived from NSCCs, which compose the majority of the CSC niche, play a key role in maintaining CSC stem-like properties and survival in response to chemotherapy. Proteomic analyses of EV cargo proteins suggested that horizontal transfer of protein synthesis/degradation machinery may be critical for CSC survival, maintenance, and plasticity. These results suggest an intriguing scenario wherein the majority of NSCCs will die in response to chemotherapy, thereby releasing EVs containing key cargo molecules capable of facilitating CSC survival and integrity. CSCs are known to have low translational activity and reduced proteasomal activity [29]. Recent studies have reported that ribosomal protein alterations can influence CSC properties such as stress resistance, self-renewal, and metastatic potential. In glioblastoma, increased expression of uS17/RPS11 and uS10/RPS20 enhanced stress-resistant CSC phenotypes. In breast CSCs, silencing eL39/ RPL39 expression was found to eliminate the ability of CSCs to undergo self-renewal and metastasis [30–32]. Our findings are consistent with these reports. Taken together, our data suggest a novel pathway mediated by NSCC-derived EVs that can modulate CSC characteristics.

A majority of studies use stem cell markers to isolate CSCs, but there are inherent limitations to this approach. First, there are no universal markers suitable for CSC isolation, and many current CSC markers are not specific for CSCs, instead being co-expressed in other normal tissues, necessitating biological validation of predicted phenotypes [16, 33, 34]. Moreover, the expression of those markers is context-dependent in that they are differentially regulated in response to environmental changes. Therefore, the establishment of a reliable and efficient CSC experimental model is critical for advancing CSC research. Here, we used the established MB49 tumor-derived sub-clones MB49 S2 and MB49 F1 [18] to respectively represent CSCs and NSCCs. MB49 BC cells are originally derived from C57BL/6 mice and they share several interesting similarities with human BC with respect to cell surface markers, sensitivity to apoptosis, and immunological profiles [35–39]. As such, the MB49 model is one of the most popular syngeneic mouse models used in BC research, allowing for the translation of in vitro findings in an in vivo setting. Therefore, these genetically-matched MB49 sub-clones not only represent a unique opportunity to study the cross-talk between NSCCs and CSCs and the consequences thereof, but also provide an excellent model for the future in vivo examination of targeted CSC therapies.

Ribosomes are subcellular cytoplasmic biomolecules composed of rRNA and dozens of proteins that primarily participate in translation [40, 41]. Recent studies have shown that ribosomes are involved in multiple biological processes, such as cellular proliferation, differentiation, homeostasis, and development of cancer [40, 42, 43]. Undifferentiated CSCs are known to maintain low levels of global translation and degradation activity, but they need to respond to dynamic alterations in the tumor environment during disease progression by rapidly regulating protein translational programs [44–46]. Maintaining the ability of CSCs to undergo self-renewal requires high translation efficiency [41, 45, 47], and inhibiting translation in CSCs has the potential to reduce their stem-like properties [48]. Our data revealed that EVs released by NSCCs contain cargos enriched in ribosomal proteins that could be taken up and utilized by CSCs to rapidly induce protein synthesis, enabling these cells to adapt to a dynamic tumor environment. This NSCC EV-mediated paracrine signaling mechanism highlights a novel potential driver of CSC plasticity, self-renewal, and chemoresistance.

Overcoming chemoresistance remains a major challenge in treating cancer patients. EVs have been long recognized as key mediators of cancer cell treatment responses. Under therapeutic conditions, cancer cells release EVs that can be transferred to neighboring recipient cancer cells, promoting their survival. For instance, cancer cells release EVs containing multidrug resistance protein 1 (MDR-1), a drug efflux pump, and treatment with those MDR-1 containing EVs facilitates the survival of various cancer cells, including prostate and ovarian cancers, acute T lymphoblastic leukemia, and osteosarcoma cells [49–52]. In addition to facilitating cargo transfer, tumor cell-derived EVs may enable chemotherapeutic drug sequestration and expulsion, thereby reducing associated cytotoxicity (53). In this study, we demonstrated that under CDDP and gemcitabine treatment conditions, NSCCs released EVs capable of transferring cargo proteins to CSCs, thereby facilitating acquired chemoresistance. Our data highlighted an interesting dynamic scenario whereby chemotherapy can eliminate the majority of cancer cells, yet before they die, those moribund cells release EVs that can support the survival of CSCs under these treatment conditions, ultimately resulting in resistance and disease progression. Therefore, the inhibition of EV release/internalization via small molecule drug treatment, and/or the targeting of specific EV cargo proteins and their pathways may hold therapeutic promise as a means of antagonizing chemotherapy-induced adverse effects.

## Conclusions

In conclusion, our study reveals a novel cell–cell communication mechanism (Fig. 5) in which NSCCs and CSCs interact via their EVs in a manner that alters CSC fate and enhanced the fitness of these cells in the context of disease progression to promote the acquisition of chemoresistance.

## Supplementary information

**Additional file 1.** **Fig. 1** Proteomic analyses highlight potential functional roles for S2-EV cargo proteins. A. Normalized abundance values for F1-EV and S2-EV cargo proteins were calculated following LC-MS/MS, and log2 fold-change (FC) values were calculated by comparing the relative abundance of a given protein in F1-EV and S2-EV samples. Green lines correspond to a |log2(FC)| ≥ 2.0, and the horizontal gray line corresponds to the arbitrary threshold for highly abundant proteins used in GO/KEGG pathway analyses. B. Top GO terms and KEGG pathways corresponding to highly abundant proteins that were preferentially enriched in F1-EVs (|log2(FC)| ≥ 2.0; relative abundance ≥ 108; n=128), as calculated using the DAVID bioinformatics database. C. Heatmaps demonstrating the relative abundance of proteins in the Proteasome, Ribosome, and Spliceosome KEGG pathways in F1-EV and S2-EV samples.

## Data Availability

The datasets used and/or analysed during the current study are available from the corresponding author on reasonable request.

## Abbreviations

BC: Bladder cancer
CSCs: Cancer stem cells
CDDP: Cisplatin
EVs: Extracellular vesicles
FBS: Fetal bovine serum
MDR-1: Multidrug resistance protein 1
MIBC: Muscle-invasive bladder cancer
MTT: Methylthiazolyldiphenyl-tetrazolium bromide
mEGF: Mouse recombinant epidermal growth factor
mbFGF: Mouse recombinant basic fibroblast growth factor
mLIF: Mouse leukemia inhibitory factor
NSCCs: Non-stem cancer cells
NMIBC: Non-muscle invasive bladder cancer
NTA: Nanoparticle tracking analysis
TME: Tumor microenvironment
