# Supplementary figures and images for: Non-stem bladder cancer cell-derived extracellular vesicles promote cancer stem cell survival in response to chemotherapy
Source: Stem Cell Res Ther. 2021 Oct 9;12:533. doi: 10.1186/s13287-021-02600-6 (PMC8502272; doi:10.1186/s13287-021-02600-6)

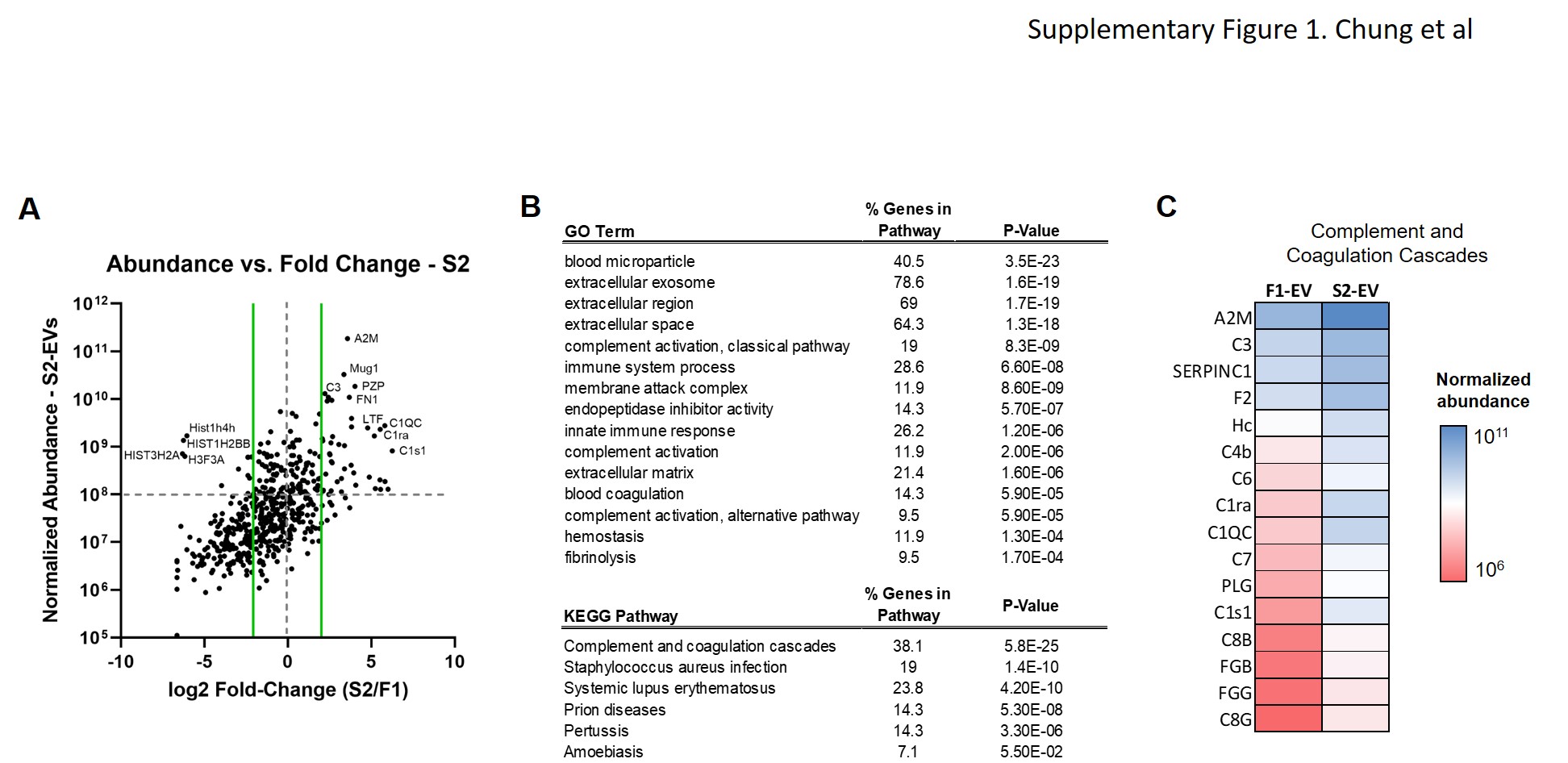

Supplement: Supplementary file 1 — Additional file 1. Fig. 1 Proteomic analyses highlight potential functional roles for S2-EV cargo proteins. A. Normalized abundance values for F1-EV and S2-EV cargo proteins were calculated following LC-MS/MS, and log2 fold-change (FC) values were calculated by comparing the relative abundance of a given protein in F1-EV and S2-EV samples. Green lines correspond to a |log2(FC)| ≥ 2.0, and the horizontal gray line corresponds to the arbitrary threshold for highly abundant proteins used in GO/KEGG pathway analyses. B. Top GO terms and KEGG pathways corresponding to highly abundant proteins that were preferentially enriched in F1-EVs (|log2(FC)| ≥ 2.0; relative abundance ≥ 108; n=128), as calculated using the DAVID bioinformatics database. C. Heatmaps demonstrating the relative abundance of proteins in the Proteasome, Ribosome, and Spliceosome KEGG pathways in F1-EV and S2-EV samples. [file 13287_2021_2600_MOESM1_ESM.jpg]
